# Proceedings of Chicago Dental Society

**Published:** 1885-03

**Authors:** 


AMERICAN JOURNAL

OF

DENTAL SCIENCE.

VOL. XVIII. THIRD SERIES—MARCH, 1885. NO. 11.

ARTICLE I.

### CHICAGO DENTAL SOCIETY.

This Society held its regular monthly meeting February 3, President A. W. Harlan, in the chair.

Dr. J. H. Woolley read a paper on the "First Permanent Molar."

### ABSTRACT OF DR. WOOLLEY'S PAPER.

There are many causes at work to produce a healthy, or unhealthy condition of the teeth under consideration, such as hereditary and pre-natal influences.

The age we live in, and the manner of life of the child, together with its inheriting a highly nervous organization, all combined, prevent a harmonious development of the first permanent molar. Neglect upon the part of the parent and dentist plays an important part in the early destruction of these teeth. Mr. Tomes' record of 3,000 extractions of teeth, one-third of them being first permanent molars, does not seem to me a fair test, as a majority of the extractions occurred among the poorer classes in hospital practice. Many dentists believe these teeth are short-lived and that the space gained by their removal prevents decay of those remaining.

About the time of the shedding of the central incisors four first permanent molars appear, and at a time when most of the temporary teeth still remain. By carelessness or inattention parents overlook these teeth, or, if they are noticed, they suppose them to belong to the first set.

The child is brought to the dentist on account of toothache. Many dentists finding the child difficult to manage, hurry through the operation, with the result that the operation is often imperfect and sometimes a complete failure.

By the extraction of the first permanent molar, it is claimed there will be room gained for the biscuspids and canine teeth to fall back, and the space gained prevents these teeth from decaying. I, as well as others, have found that the removal of these teeth made very little difference in regard to the decay of the remaining ones; also that the anterior teeth seldom move back, but forward. Here followed a quotation from Dr. Arthur supporting this position.

It may be necessary to remove these teeth in some cases, because of decay, but it is a mistake to remove them to correct irregularities. It is the opinion of many of the best authorities, that irregularities are not caused by lack of room in the jaw. Quotations from Dr. Kingsley and Mr. Tomes were introduced to substantiate this view. In a majority of mouths in cases of irregularities, I have found by measurement of the models of these mouths, that there was room enough to bring the teeth into position without the extraction of the first permanent molar. The extraction of these teeth causes a depression in the face and a lost of individual expression and also effects the voice.

In a private letter from Dr. A. G. V. Black he says: "One point that I have especially urged is the office of the first permanent molar in holding the jaws in position during the process of the shedding of the temporary teeth. This point so often overlooked I deem very important. These teeth take their position before the beginning of the shedding, and while the antagonization is otherwise broken up they the jaws in position and prevent any twisting of them by the powerful masseter and temporal muscles, a thing that is very liable to occur if this support is lost by the removal of any one of them before the twelfth year molars have come into position.

If these teeth could be kept until the child has arrived to its teens, their chances would be good for permanent retention." He further says "my study of the comparative liability of the teeth to decay at the different periods of life, shows clearly that the first molars are attacked in the first two or three years after eruption much oftener than any other teeth, but in after years they are attacked less often then the second.

If both the teeth are in a fair condition at the age of fifteen the chances for the first are better than the second. Decay occurs on the posterior surface of the second molar much oftener than in the same position on the first, and is much more difficult to treat."

Many claim the first permanent molar will last but a few years. Experience in the treatment of these teeth for a number of years, has taught me if they are watched with the same zealous care as others, they in all probability can be saved.

In making an examination the coffer dam should always be used, as the crowns of these teeth can then be examined more critically. If the temporary molar is in close contact with the first permanent molar, the distal surface of the former should be separated therefrom with a safe-sided file, and if the mesial surface of the latter is superficially decayed, the decay should be removed.

The tipping or lapping of the crowns of the second and third molars is occasioned by the loss of tissue, as a result of the extraction of the first.

Great care is necessary in the treatment of these teeth. In large cavities my mode for the last few years has been different from that previously followed.

In deep cavities that are sensitive, with approximate exposure of the pulp, some of the cements should be used for filling. Flow the cement over the floor of the cavity and after hardening fill the remainder with the same material mixed harder. When the cement begins to wear away, if the tooth has caused no trouble, remove a portion of the cement and fill with tin and gold combined or with whatever material is best suited to the case.

I believe these teeth more susceptible to thermal changes when metallic fillings are used than are other teeth.

In conclusion, I believe that when these teeth, particularly in the first two or three years after their eruption, are studied and properly cared for; the universal verdict will be it will be the fault of the dentist and not of the tooth if they are not saved.

DISCUSSION.

*Dr. Noyes.*—It seems to me that the conclusions of the essayist are just, and that the first molars eminently deserve the strong pleadings for their care and preservation which we have frequently heard of late. They are, normally, the largest teeth in the mouth, with the most perfectly and strongly developed roots. They have the largest masticating surfaces and stand in the places where the largest amount of chewing is most easily done. It seems, therefore, that they were intended to be the most important of the molar teeth.

The question of their preservation or removal should be carefully considered in each case upon its merits, and the judgment should not be warped by a predisposition derived from the general statement wich we sometimes hear expressed, that "it is better in most cases to remove them." or "if the teeth are at all crowded and of poor quality it is almost always better to extract them," and others to the same purport. If they cannot be preserved, I believe less harm will result from their loss, and equal advantages will be usally gained if their extraction is delayed (as it can usually be by taking care of them) till the bicuspids and second molars are in their places and have acquired their normal articulation and their roots become fully formed. Nature puts the first molars in their placcs, strongly and fully developed, and meeting each other squarely, before the temporary molars and cuspids begin to loosen, and during several years they furnish the only adequate support and resistance to the action of the powerful jaw muscles, permitting the bicuspids and second molars to attain their proper length and perfect their roots before they are called upon to do full work. If the first molars are absent during this time, the undue force brought to bear upon half grown teeth may drive them out of their places with varying degrees of irregularity, or they may be prevented from attaining their proper length, and the too close approximation of the jaws may cause the upper front teeth to project forward, or in some other way mar the features. You have all doubtless seen some deplorable results that might with probability be referred to this cause.

*Dr. Ames.*—One point of the paper as I understand it, is that the first permanent molars being extracted, the teeth anterior to it would invariably fall forward. As a natural result of the occlusion of the bicuspids I have found that the second superior bicuspid will move backward, but I have never observed any such results as referred by the essayist. In my early practice I extracted first permanent molars, expecting beneficial results, but discarded that practice some few years since. They are, anatomically, the best teeth in the mouth, and the cases are very rare in which these teeth cannot be retained to answer a useful purpose. The essayist remarked also that the teeth of the young are more sensitive to thermal changes. This, of course, is naturally the case; but I think the pulp of a tooth which has recently erupted will recover from more irritation than that of an adult tooth. The blood vessels are more numerous and are not so apt to become strangulated at the apical foramen as a result of irritation.

*Dr. Brophy.*—It appears to me that a great deal of unnecessary time is spent in the filling of these teeth. It has been my practice for some time to introduce cement and keep it there until it wears away, put in some more, and so treat such cases until patients have arrived at an age, where it will be worth one's while to introduce gold. In treating a first permanent molar at seven, ten or twelve years old, I would prepare the cavity as well as I could and would, as a filling material, use oxyphosphate of zinc. It doesn't require much pressure to destroy a pulp. Fill the cavity as well as possible, and subsequently when the cement has worn down somewhat, cut out the margins and put in some oxchloride instead of oxyphosphate, because the latter is much harder, and consequently better, provided it is kept dry. I find this to be an excellent practice. In teeth where the pulp is partly or nearly exposed, I prefer to introduce gutta-percha rather than the chances of destoying the pulp by introducing any of the cements. I think it is the best way of all to fill teeth of that kind. Teeth are like apples in one respect. One bad one will spoil the whole lot, and this is particularly true with regard to the first permanent molar, which contracts decay from the adjacent temporary. Children in a great many cases dread dentists, and this dread is due to unnecsssarily long and painful operations. I don't think amalgam should be used in the first permanent molars of a child.

*Dr A. E. Matteson.*—I have a case in hand at the present time which has caused me considerable solicitude, and is undoubtedly the result of neglecting the sixth year molars. It is that of a miss of fourteen years, of highly nervous organization. The crowns of the inferior left and superior right sixth year molars were decayed to the margin of the gum, the pulps of both were hypertrophied to the extent of nearly filling the cavity of decay. Owing to the pain caused by masticating upon these during the time of the eruption of the twelfth year molars, the proper articulation has been lost in the ten anterior teeth in each jaw, and are not occluding by 3-16 of an inch, the twelfth year molars preventing it. Have extracted the offending sixth year molars, and would like to hear any suggestion as to the best method of correcting the irregularity.

*Dr. Pruyn.*—I would like to hear Dr. Allport answer this question.

*Dr. Allport.*—In answer to the question propounded by Dr. Matteson, I will say that in order to answer it intelligently it would first be neccssary to know how near the anterior teeth come together, as well as several other surrounding conditions. But assuming that they come within just 3-16 of an inch, as stated by Dr. Matteson, and that the molar teeth mentioned were the only cause that prevented a perfect occlusion oi the anterior teeth, I think I might possibly cut off the grinding surfaces of the molars as far as I could without endangering the vitality of the pulps, or until the anterior teeth came nearly together. Then with a plate, or with wedges, I would move these teeth in their sockets to excite such a development of the jaw as would produce a perfect occlusion of the remaining teeth.

In regard to the first permanent molar teeth—-the subject of the paper—I will say that if I were asked to state what one thing in my opinion in dental practice required the most enlightened judgment, I should unhesitatingly reply, under some circumstances, the proper treatment of the first permanent molars.

That they should, if possible, be saved when the retention will not endanger the health of the patient or impair the usefulness of the other teeth does not admit of a doubt. An organ should not be sacrificed so long as the health of the individual is not endangered or the usefulness of other organs of the economy impaired. This is a good rule in general surgery and should hold good in dentistry, for dental surgery is but a part of general surgery.

No reasoning would as thoroughly convince me of this, as have the many years of observation I have had, in examing the mouths of my own patients as well as those of other dentists who have followed widely different views in practice regarding the treatment of these teeth, some attempting to save nearly all of them and others giving largely to extracting them. If the teeth and jaws are harmoniously developed, the teeth all well formed and dense in structure, the extraction of these teeth would certainly be bad practice; but unfortunately these conditions do not always exist, and our practice should be aimed to secure the best results possible under the existing conditions.

To say that these teeth should always be retained, if possible, as intimated by the essayist, is a very broad assertion, for it will be frequently found that greater injury will be caused to the other teeth by their retention than the patient would suffer by the loss of them. So it is not alone the possibility of saving the teeth that is to be considered in determining what should be done with them, but the amount of injury likely to occur consequent upon their retention. "The greatest good to the greatest number," in this as in other things, is a wise maxim to follow.

The condition of the surrounding teeth, as I have previously said, is an important factor. If the teeth are soft and predisposed to decay, and if in addition to this they are crowded, it is often necessary to remove more or less of the proximate surfaces when decay has attacked these locations. Then from imperfect operations or a lack of cleanliness decay often takes place around these fillings and they have to be removed; more or less of the surface is again cut away and by the time the patient is 35 or 40 years old, less grinding surface will be left and the mouth will not be in as good a condition as through these teeth had been extracted at the proper time.

There is no use in saying that this is not true, for old and good practitioners of close observation *know* that it is a fact.

*Dr. Harlan.*—How about these teeth when they have lost their pulps?

*Dr. Allport.*—With regard to the question just asked by Dr. Harlan, "How about these teeth when they have lost their pulps?" I will say, during the last few months we have seen a good deal in the dental and medical journals about "Dead teeth in the jaws," and some good things have been said both for and against the practice of retaining them, and some other things had better not have been said at all. Whatever of truth has been said against the practice in general is eminently so in regard to young patients, for whatever injury is done must be consequent upon the gases generated by the decomposition of the organic tissue within the pulp canal and the dentine, by septic influence or pressure upon the surrounding tissues. This may be produced at the apex of the root, or possibly through the cementum upon the peri-cemental membrane.

The younger the subject the greater the amount of organic tissue in the dentine, and consequently a greater amount of these gases will be formed by the processes of decomposition, and the younger the subject the more certain will it be for deleterious effects to follow. It is true these roots may be filled, which will go far towards preventing these conditions; but the greatest skill in this direction will not always prevent after trouble.

An alveolar abscess is not the only cause that may call for the removal of such teeth, and the neural troubles, urged as a cause for their extraction, seldom arise from acute inflammation or from alveolar abscess.

The diseases of the eye and ear mentioned in the discussion in the *Medical Record*, and other journals, as the result of retaining these teeth in the jaws, is not altogether improbable, because the low form of irritation so frequently produced in the peri-cemental membrane by the gases generated within the root canal, or in the tubuli of the dentine, is the most fruitful source of reflex nervous conditions associated with the teeth; and many times these reflex troubles arise from a state of irritation so low as to be very difficult of detection and quite likely to escape observation, even after a most careful examination, as has been proved by the cure of the diseases on the extraction of the teeth only suspected of being the harbor of the mischief caused; and if these conditions are produced in older subjects by the retention of these teeth, we should be cautious how we keep them in the mouths of our young patients.

*Dr. Pruyn.*—At what age would you extract these teeth?

*Dr. Allport.*—Unless there was some good reason for their earlier removal, I should with inexpensive fillings try and keep them until the patient was fifteen or sixteen years old, or until such time as the natural occlusion (inter-locking) of the teeth was perfectly established.

*Dr. J. S. Marshall.*—It was my privilege for many years to practice in the same city in which that prince of dentists, Dr. Amos Westcott, spent the greater part of his professional life, and I observed that it was his practice to extract badly decayed first permanent molars, rather than to devitalize the pulps and perform extensive operations upon them. The result of this practice —when performed before the eleventh year—I often noticed was in many cases to prevent approximal decay upon the side of the arch from which the tooth was removed. To illustrate—and this is one from a great many that might be cited—a young gentleman had the first permanent molars extracted upon the left side when about ten years of age; at twenty-seven he had not a single approximal cavity in the teeth of that side, while upon the right side the first permanent molars had been allowed to remain, and I found the bicuspids and first and second molars all had approximal fillings in them.

I have seen the same condition of things in the mouths of patients who had been under the professional care of other noted practitioners. As a consequence of this observation, backed by my own experience, I am now in the habit of extracting these teeth if I find them very badly decayed and associated with exposed or dead pulps, and consider it good practice to do so. Of course I would not extract them under all and any circumstances; judgment must be exercised, and the present conditions as well as the future possibilities taken into considerations. It has been my experience that when these teeth were removed before the eleventh year, the second molars have come forward and taken the place very nearly of the extracted teeth, and their loss has hardly been recognizable. I have no hesitation under certain circumstances, viz.: when the teeth are soft, badly decayed and much crowded in the arch, of extracting all four of the permolars as a prophylactic measure. Such treatment gives space for the remaining teeth, places them in a better condition to be kept clean, and consequently caries is less likely to appear upon the approximal surfaces.

*Dr. Crouse.*—I had thought I would not speak on this subject and do not know now as I ought to say anything. For, in doing so I must take radical ground against some who have spoken who are men of reputation, and such differences must have a tendency to mislead the young practitioners and cause them to think there are no established facts in dentistry. There are established principles and there are the facts which should act as a guide in helping us to decide how to treat even the sixth year molars.

The last gentleman on the floor (Dr. Marshall) cites a case where the first molar had been extracted and states that the approximal surfaces of the rest of the teeth on that side were all sound requiring no filling, while on the other side the first molar was let remain and all the teeth on that side required constant filling. He then asks us if that is not sufficient proof of the soundness of the practice of extracting this tooth. Why I could cite a hundred cases coming under my own observation, where in consequence of the early extraction of the sixth year molar, the usefulness of the balance of the teeth on that side as masticating organs where, if not wholly, to a very great extent, destroyed. The loss of the first molar allowed the bicuspid, not as urged by the essayist, "to go forward," but to lean backward, and the second molar to pitch or lean forward to so great an extent, many times, as to allow the tops to touch each other, thus destroying the natural antagonism, and instead of the teeth above and below matching into each other they only touched here and there on the extreme points. The mal-position thus cause made it next to impossible to save the second molar and bicuspid. With the first molar and its socket gone, and the tops of the two teeth touching, the large space is left below, and with the decks of both teeth exposed even to a considerable portion of the cementum, it will be impossible, many times, to prevent caries from going on in this pocket by any amount of filling. Too often have I seen the usefulness of the teeth destroyed by the loss of the sixth year molar in the manner I have already described, to advocate such a practice. When I have seen God's handiwork thus multilated and an irreparable injury done the patient by the hand of what was called a dentist, and then see in my next journal somebody advocating the extracting of the sixth year molar, it has made my blood boil, knowing that such advice would cause many a young practitioner to destroy or greatly injure what would, if otherwise treated, have been a complete and perfect masticating apparatus. If I cannot do better, I prefer to keep the roots filled and keep them as long as possible to make the other teeth keep their normal position.

We have heard also about the gases in these sixth year molars from a practitioner of reputation, Dr. Allport, and have been told that when the pulp is dead the sixth year molar should be extracted on account of its gases in the tooth being very injurious, ect. Grant that a tooth with decomposition and gases in the roots does do injury; why extract the sixth year molar under such conditions sooner than any other tooth with a dead pulp? Treat them and get rid of the gases, then fill the roots properly, and I will warrant no injury will come from them in that condition. There is a class of cases, however, in regard to which I am more in doubt, that is, when the pulp has been lost at a very early age before calcification has nearly completed its work; where even the end of the root from this early death of the pulp has not been completely formed, so that the entire end is open, making it almost impossible to fill them properly or perfectly. If there are any cases where I would extract, it would be these, and I would not in these cases unless I was obliged to. I would try and keep them until the other teeth had taken their natural antagonism. If I were obliged to lay down any universal rule on this subject, I should say *never* extract the sixth year molar. Such a rule would be far better than what has been advocated here to-night, viz, to generally extract, or to extract even any considerable number. I should say *seldom* should the sixth year molar be extracted. I was glad to see the essayist take the radical ground he did against extracting them. The proportion of honest practitioners who extract this tooth, as a general rule, is growing less and less, and thank God for it.

Now in regard to the case spoken of by Dr. Matteson. If I have anything like the correct idea of the case, I should expect, if the patient is young, that time would remedy, to a considerable extent, the deformity. It is perfectly natural for the teeth to articulate. You will see this illustrated in a practical way, if you will cap some of the teeth, or allow a plate to be worn which will keep part of the teeth from striking, how soon the others will elongate. I should be very slow to grind off three-sixteenths of an inch from each of the second molars. It occurs to me that anything like that proportion of grinding would destroy too much tooth; in fact, I don't think it could be successfully done. I should expect time to work wonders in that case.

*Dr. G. W. Nichols* was delighted that a subject had at last been reached on which there was a similarity of views. As regards the sixth year molar he wished that he had never seen one. It had caused him unlimited trouble, and he thought it was a serious commentary upon modern civilization, parentage, life, knowledge and practice. Young married couples rushed into parentage without the least idea of the consequences entailed and attending the sixth year molar. He maintained that no couple should get married, and that no minister should perform the ceremony without seeing that they were posted with regard to the temporary teeth. He was puzzled every day of his life about these teeth, what to do with them. Trying to save them had shortened his life. If there was a certain class of the community that should understand the condition of these teeth, it was the family physicians. Ignorance on the part of parents might be excused, but a lack of knowledge on the physician's part could never be. Nineteen out of every twenty graduates of medical colleges *know nothing about the sixth year molar.* Physicians should be able, and taught to consider it their duty, to inform parents of the possible decay of these teeth, and it should be the duty of college faculties to see that no one graduates who is in ignorance about the sixth year molar. The question of extraction or retention of such teeth was a hard one to decide, but the longer he lived the more he would endeavor to save the last one of them, but the last one he would extract, if necessary.

*Dr. Woolley* in closing the discussion, said: If we imbue our patients with the necessity of preserving these teeth, speak to them intelligently, and secure their co-operation in our labors, we shall accomplish better results.

On motion the society adjourned.—*Archives of Dentistry.*

